# The Relationship Between Hippocampal Volume and Activity Diversity

**DOI:** 10.1093/geroni/igaa057.2249

**Published:** 2020-12-16

**Authors:** Emily Urban-Wojcik, Soomi Lee, Susan Charles, David Almeida, Richard Davidson, Stacey Schaefer

**Affiliations:** 1 University of Wisconsin-Madison, Madison, Wisconsin, United States; 2 University of South Florida, Tampa, Florida, United States; 3 BSS, Irvine, California, United States; 4 Pennsylvania State University, University Park, Pennsylvania, United States

## Abstract

The hippocampus, implicated in learning, memory, and spatial navigation, is one of the few brain structures that demonstrates neurogenesis across the lifespan. Hippocampal volume (HV), then, may be a marker of exposure to and engagement with novel events and environments, which may in turn be related to cognitive functioning. The present study examined the relationship between HV and activity diversity (AD), which characterizes the range and evenness of participation in daily activities. In 52 participants who completed the daily-diary and neuroscience projects of the Midlife in the United States Refresher study, greater levels of AD across an 8-day period were related to greater HV averaged across the left and right hemispheres when adjusting for overall brain volume, total activity time, time between projects, and relevant sociodemographic variables, b=1128mm3, t(43)=2.54, p=.015. These findings may point to a mechanism through which AD has been related to better cognitive and mental health outcomes.

